# Plasmonic Nanocubes with a Controllable “Crescent Arc” Facet: Tunable Hotspot Engineering for Highly Reliable and Sensitive SERS Detection

**DOI:** 10.1021/acs.analchem.4c05334

**Published:** 2024-10-17

**Authors:** Ting Wang, Jinchao Wei, Zehua Cheng, Mai Luo, Liang Zou, Lele Zhang, Mei Zhang, Peng Li

**Affiliations:** † State Key Laboratory of Southwestern Chinese Medicine Resources, School of Pharmacy, Chengdu University of Traditional Chinese Medicine, Chengdu 611137, China; § State Key Laboratory of Quality Research in Chinese Medicine, Macau Centre for Research and Development in Chinese Medicine, Institute of Chinese Medical Sciences, University of Macau, Taipa, Macao 999078, China; ∥ School of Food and Biological Engineering, Chengdu University, Chengdu 610106, China

## Abstract

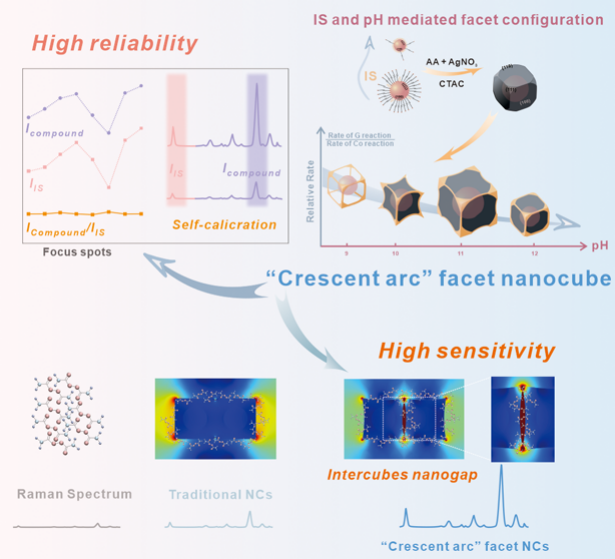

The fine control of the nanogap and morphology of metal nanoparticles (NPs) has always been an obstacle, hindering the development and application of surface-enhanced Raman scattering (SERS) quantitative detection. Here, Au/4-mercaptobenzoic acid@Ag@Au–Ag bimetal core–shell nanocubes (NCs) with a “crescent arc” facet (C-Au/4MBA@Ag NCs) as a highly reliable and sensitive surface-enhanced Raman scattering SERS substrate is proposed for the first time. The bifunctional internal standard (IS) molecules (4MBA) govern the morphology of metal shells to maintain cubic configuration and provide calibration for SERS signals’ flotation. In parallel, the controllable curvature of the C-Au/4MBA@Ag NCs is directly modulated by adjusting the relative rates of the galvanic replacement and co-reduction reaction, which generates a controllable interparticle nanogap to offer large depositing spaces for analytes and improve authoritative SERS signals’ enhancement. The proposed C-Au/4MBA@Ag NCs exhibit an enhancement factor of up to 4.8 × 10^10^ and contribute to the ultralow RSD (7.9%). These C-Au/4MBA@Ag NCs also enable the detection of hazardous pesticide residues such as methamidophos and thiram in herbal plants with a complex matrix, with an average detection accuracy of up to 96%. In summary, this study achieves a fine control strategy of the “crescent arc” surface for improving SERS performance and explores the practical application potential for accurate and sensitive Raman detection of hazardous substances.

## Introduction

Surface-enhanced Raman scattering (SERS) has been a research hotspot due to its powerful optical properties and extensive applications in various fields.^1,2^ SERS is a rapid, label-free, and nondestructive analytical technique for amplifying the vibrational fingerprint spectrum to detect molecules in solids,^3^ liquids,^4^ and even gases.^5^ Compared with other rapid detection methods, such as fluorescence spectroscopy,^6^ electrochemical detection,^7^ etc., it offers significant advantages in ultrahigh sensitivity, surpassing photostability, and powerful multicomponent detection.^8,9^ The signal amplification by SERS mainly arises from localized electromagnetic fields (EMFs), referred to as “hotspots,” and the chemical interaction between probe molecules and plasmonic nanoparticles (NPs).^10,11^ However, EMFs are affected by the configuration, size, and distribution of metal NPs, which has been a stumbling block on the way forward for accurate trace detection by SERS. Researchers have made great efforts in fabricating uniform and highly sensitive SERS substrates, such as microfluidics,^12^ electroplating methods,^13^ superhydrophobic devices,^14^ and nanogap fabrication,^15^ etc. Besides this, “hotspots” are affected by the quantity and size of spikes on metal NPs. Various anisotropic Ag or Au NPs with sharp corners and edges, including nanocubes (NCs), nanostars (NSs), nanoflowers (NFs), and urchin-shaped NPs, have been fabricated to provide strong EMFs.^16−20^ According to the “lightning rod effect,” electrons are squeezed to the tip by an external electromagnetic field to increase the charge density, thereby forming a strong EMF at the sharp corner.^21,22^ As a result, the number and configuration of spikes should be particularly strictly controlled to reduce the impact of the substrate itself on reliability. But ensuring the spikes’ controllability is also a vital issue to be solved.^23^ NCs with intrinsic edges and corners appear to be stable and excellent amplifiers for Raman signals. There have been some literature reports that NCs have an exceptional signal amplification effect as an SERS substrate. Still, it is worth noticing that the corners and edges of NCs tend to be round rather than sharp, which weakens the enhancement effect.^24,25^ To make the best use of this advantage of NCs regarding an inherent number of edges and corners, NCs with customizable size and sharp edges and corners to enhance Raman signals would be the key issue worth exploring.

A recent work stated that NCs with concave configurations have high-index facets, surface cavities, and sharp edges and corners, most of which exhibit electrocatalytic properties.^26^ In theory, concave configuration can be more beneficial for the enhancement of EMFs and exhibiting higher SERS activity than ordinary NCs due to interparticle nanogaps from surface cavities and sharp edges and corners.^27^ However, most studies focus only on the electrocatalytic properties of these concave NPs instead of systematically studying their SERS performance. The concave NCs mentioned in the few studies on SERS have limitations in reliability and sensitivity due to a lack of self-calibration ability and a multimetal nanostructure.^28,29^ We are strongly inspired by these reasons to further explore the controllable synthetic mechanism of concave core–shell NCs as well as the relationship between the curvature of concave NCs and SERS performance.

Here, we reported Au/4-mercaptobenzoic acid@Ag@Au–Ag bimetal NCs with a “crescent arc” facet (C-Au/4MBA@Ag NCs). Our study revealed that bifunctional internal standard (IS) molecules could govern the morphology of the Ag shell and assist in improving reliability. Moreover, the galvanic replacement and co-reduction reactions could provide a straightforward approach to fabricating pointed and controllable “crescent arc” facet NCs. The relative rate of these two reactions was controlled to change the curvature of C-Au/4MBA@Ag NCs as well as the size of the interparticle nanogap. Experimental results revealed that the 4MBA molecules, serving as IS and calibrating the SERS signals’ flotation to boost reliability, can alter the configuration of the shell. Furthermore, the “crescent arc” facets generate precisely regulated nanogaps between nanoparticles, providing large depositing spaces for analytes and “hotspots” to enhance authoritative SERS signals. Utilizing the proposed dual functional C-Au/4MBA@Ag NCs, we conducted precise SERS quantitative analysis using crystal violet (CV) as the probe molecule. Finally, C-Au/4MBA@Ag NCs offer exceptional reliability and sensitivity simultaneously, making them ideal for the trace detection of hazardous pesticides in herbal plants.

## Results and Discussion

### Principle, Preparation, and Characterization of C-Au/4MBA@Ag NCs

#### Working Principle of C-Au/4MBA@Ag NCs

**Scheme 1:**
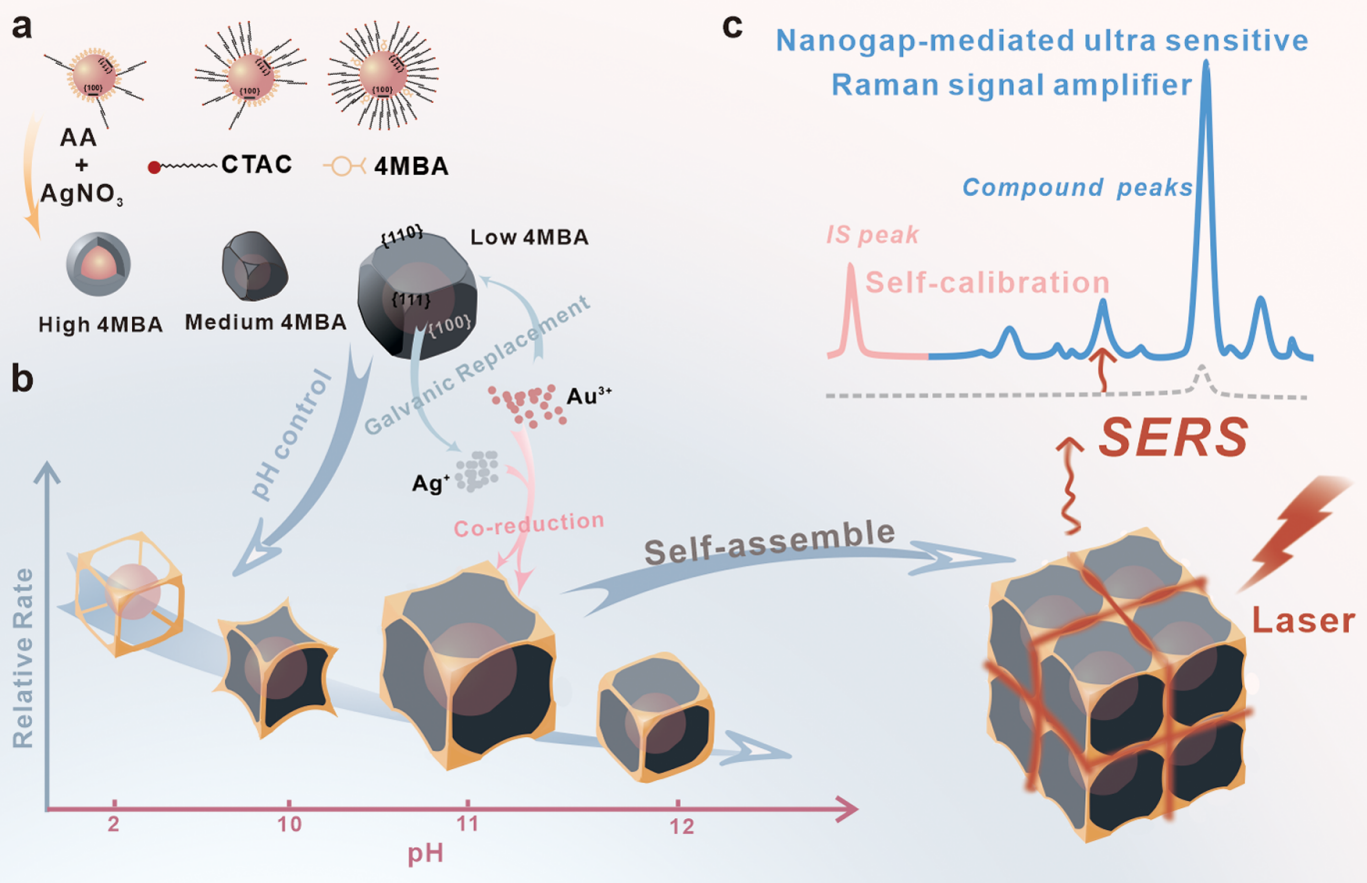
Schematic Diagram of the Synthesis and Working Principle of Curvature-Controllable NCs with IS for SERS Detection: (a, b) the Synthesis Process of NCs with IS (a) and Curvature-Controllable NCs with IS (b), and (c) the Working Principle of Curvature-Controllable NCs with IS for SERS Detection

The procedure for synthesizing C-Au/4MBA@Ag NCs involves two crucial steps, as illustrated in Scheme 1. Initially, Au/4MBA@Ag NCs are produced using the Au core as seeds. The selective deposition of Ag atoms on Au NPs is achieved by regulating the coordination with 4MBA and the reduction ability of ascorbic acid (AA; Scheme 1a). Subsequently, the galvanic replacement reaction (GRR) between the Ag shell and the Au^3+^ precursor occurs on the side facets of Au/4MBA@Ag NCs.^27^ Under alkaline conditions, AA is converted to a stronger reducing agent (HAsc^–^) by NaOH, which can simultaneously reduce Ag^+^ and residual Au^3+^ and then deposit them on the {110} and {111} facets.^27,30^ Thus, by regulating the pH of the solution, the relative rate of the GRR and co-reduction reaction (CRR) can be manipulated to produce C-Au/4MBA@Ag NCs with diverse curvatures (Scheme 1b). In the subsequent section, we discuss the specific pH effect on curvature and its growth mechanism in detail. The C-Au/4MBA@Ag NCs have eight sharp corners and “crescent arc” facets, thus generating controllable interparticle nanogaps to offer large depositing spaces for analytes and “hotspots” to improve authoritative SERS signals’ enhancement effect. On the other hand, 4MBA leads to the C-Au/4MBA@Ag NCs exhibiting inherent SERS signals, which calibrate the SERS signals’ fluctuation and improve reliability (Scheme 1c).

#### Investigation on the Growth Mechanism of Au/4MBA@Ag NCs Regulated by CTAC and 4MBA

**Figure 1:**
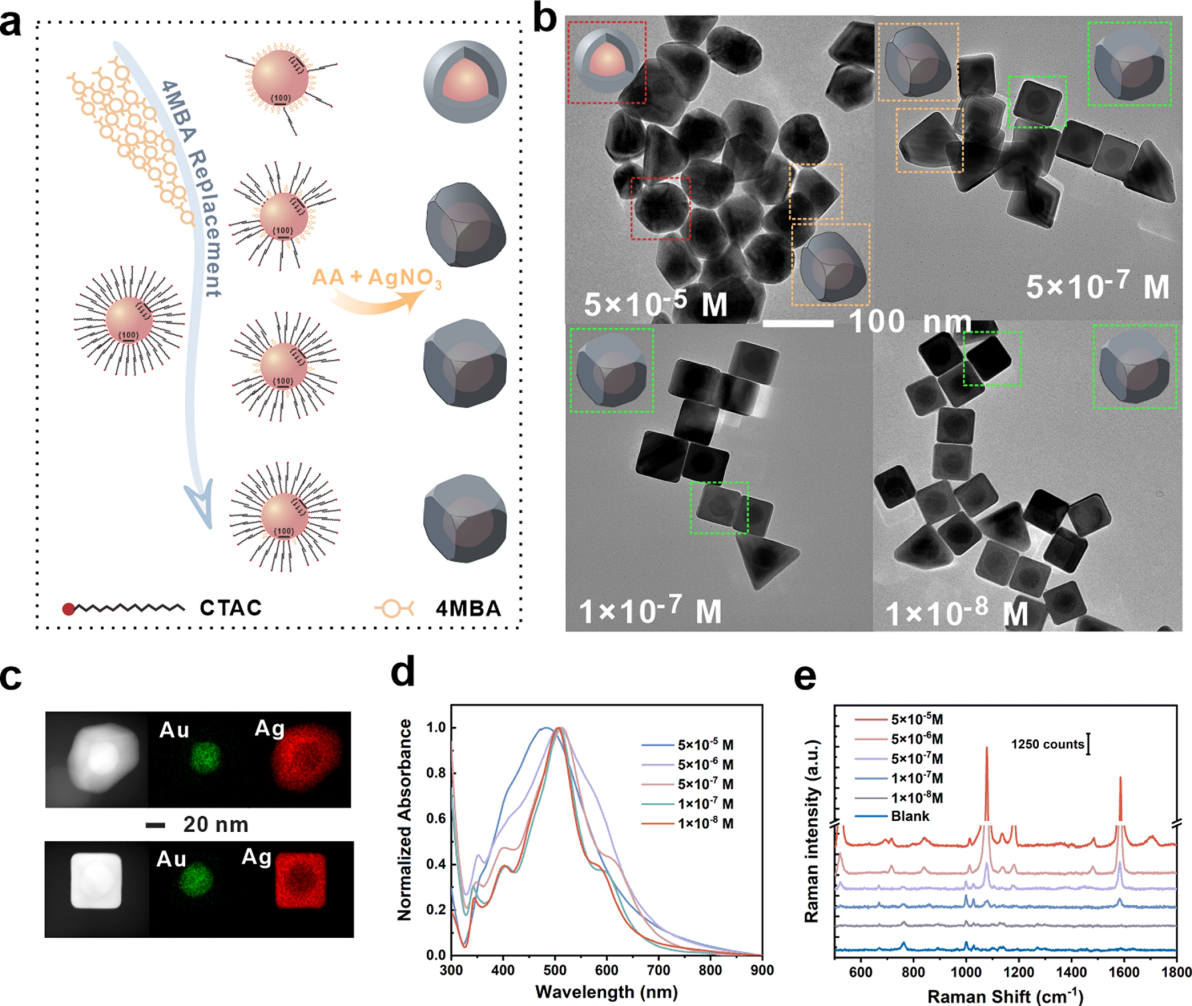
(a) Schematic diagram of NP configuration and corresponding 4MBA concentration. (b) Typical TEM images of the Au/4MBA@Ag NPs with different amounts of 4MBA. (c) STEM-EDS elemental mapping results of Au/4MBA@Ag NPs with different configurations. (d) UV–vis absorption spectra of the Au/4MBA@Ag NPs with different amounts of 4MBA. (e) Typical SERS spectra of the Au/4MBA@Ag NPs with different amounts of 4MBA.

35 nm spherical Au NPs were employed as the core in our work (Figure S1), and the seed growth method was used to synthesize Au/4MBA@Ag NCs.^31^ Then, a series of NPs were synthesized with varying concentrations of 4MBA (Figure 1a), and their corresponding transmission electron microscopy (TEM) diagrams are shown in Figure 1b and Figure S2. The NPs with 5 × 10^–5^ M 4MBA have a highly heterogeneous configuration, primarily comprising spherical and trapezoidal NPs, and the excessive addition of 4MBA results in aggregation between Au NPs, which leads to the broader ultraviolet (UV) spectrum in Figure S3. The proportion of NCs increases significantly as the concentration of 4MBA decreases, and most NPs have cubic configuration below 1 × 10^–7^ M 4MBA. The energy dispersive X-ray spectroscopy (EDS) elemental distribution mapping of Au/4MBA@Ag NPs with 5 × 10^–5^ M and 1 × 10^–7^ M 4MBA in Figure 1c indicates the successful formation of a core–shell structure. Moreover, the UV–visible (UV–vis) spectra of Au/4MBA@Ag NPs with various concentrations of 4MBA also reveal that as the concentration of 4MBA decreases, the width of the UV–vis absorption peak gradually becomes narrow, indicating a tendency toward homogeneous NP size (Figure 1d). At the same time, the NPs gradually unify from different shapes into cubic. The UV–vis absorption peak red shift from 480 to 508 nm and two higher-order plasma modes of 4MBA@ NCs are observed at 340 and 388 nm, which predict the successful synthesis of Au/4MBA@Ag NCs. The CTAC on the surface of Au NPs amplifies the rate of Ag growth on the {111} facets of Au seeds in contrast to {100} facets, ultimately leading to a cubic structure.^31^ However, the sulfhydryl group in 4MBA (IS) exhibits a strong affinity for the surface of Au NPs, resulting in the formation of a Au–S bond. It appears that the random replacement of CTAC by 4MBA has broken the law of the growth rate of Ag on all facets of Au NPs, resulting in NPs with various configurations.

The stable IS signals can significantly enhance the reliability of the SERS quantitative detection. Nevertheless, the absolute SERS signal intensity of 4MBA is determined by both the concentration and the thickness of the Ag shell. Au/4MBA@Ag NCs with varying thicknesses were synthesized by adjusting the amount of AgNO_3_ (Figure S4), and the corresponding UV–vis spectrum is shown in Figure S5. The medium-thickness (∼5 nm) Au/4MBA@Ag NCs was applied to investigate the IS SERS intensity initially. Figure 1e reveals that Au/4MBA@Ag NPs with 5 × 10^–5^–1 × 10^–7^ M 4MBA exhibit stable 4MBA characteristic peaks (1076 and 1585 cm^–1^), while with 1 × 10^–8^ M 4MBA, they do not provide valid IS information (Figure S6). Notably, Au@Ag NCs without 4MBA display CTAC characteristic peaks at 762, 1000, and 1027 cm^–1^, which are absent in NPs with high concentrations of 4MBA. In contrast to the NPs with high-concentration 4MBA, the NPs with low-concentration 4MBA show 4MBA and CTAC characteristic peaks simultaneously. These findings in turn support our hypothesis that 4MBA replaces CTAC on the surface of Au and impedes the synthesis of cubic shells. 1 ×10^–7^ M 4MBA represents an ideal choice, since it affords both a stable IS SERS peak signal and uniform configuration, thereby warranting its selection as the IS concentration for future research.

#### Synthesis Mechanism of C-Au/4MBA@Ag NCs with Various Curvatures

**Figure 2:**
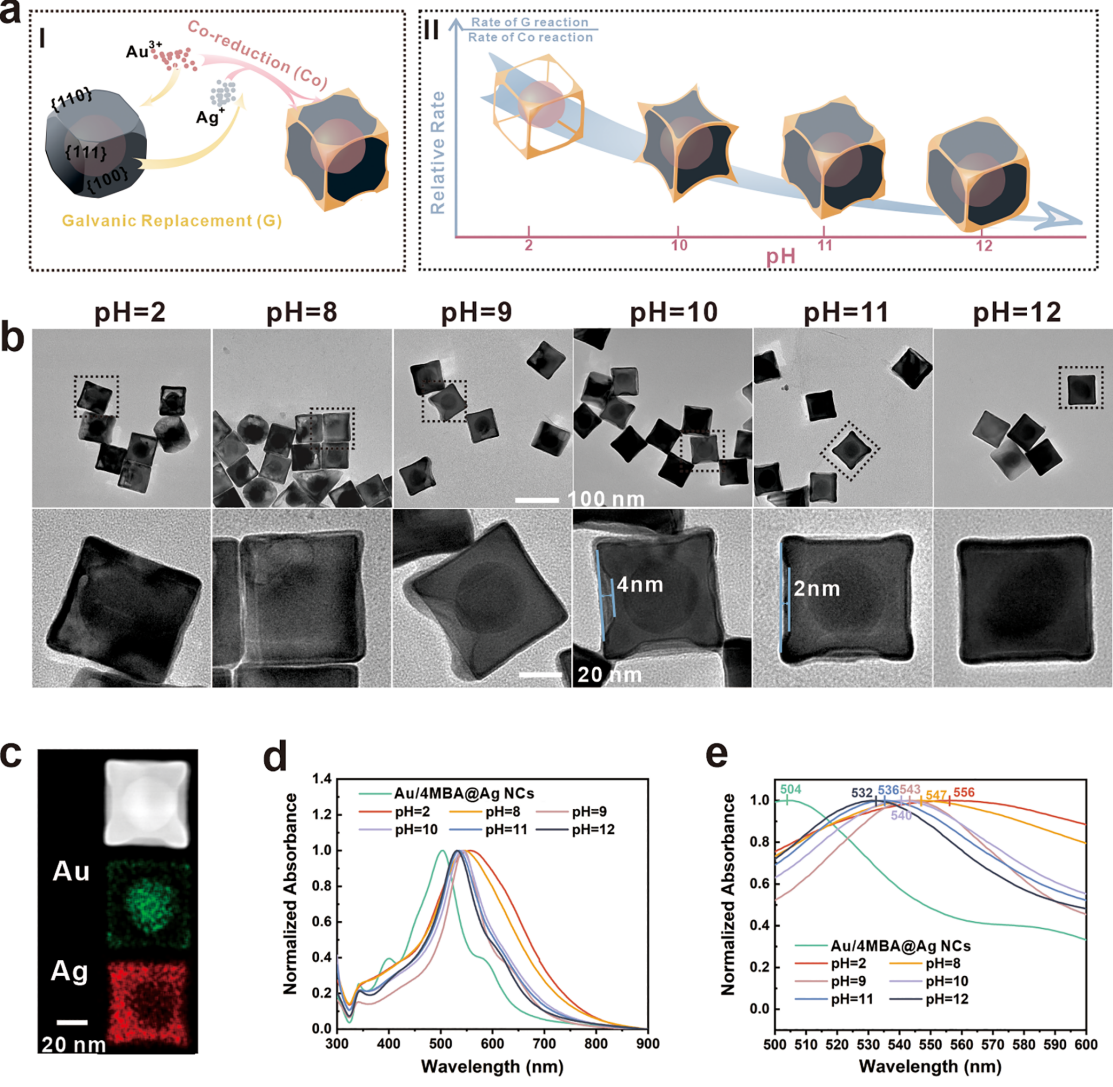
(a) Schematic diagram of the synthesis principle of concave NCs with controllable curvature. (b) Typical TEM images of the C-Au/4MBA@Ag NCs with different pH conditions, including pH 2, 8, 9, 10, 11, and 12. (c) STEM-EDS elemental mapping results of C-Au/4MBA@Ag NCs. (d) UV/vis absorption spectra of the Au/4MBA@Ag NCs and C-Au/4MBA@Ag NCs with different pH conditions. (e) Enlarged view of the absorption spectra regarding the blue shift of the C-Au/4MBA@Ag NCs.

According to the description in Scheme 1, C-Au/4MBA@Ag NCs with different curvatures could be obtained by simply adjusting the pH value of the solution. The pH of the solvent governs the relative rate of GRR and CRR, thus directing the configuration of the ultimate NCs. The CRR starts to participate in the final configuration sculpting with increasing pH, and its presence ensures the integrity of the {111} and {110} facets in the cubic shell. The reason is the preferential deposition of Au^3+^ and Ag^+^ on {111} and {110} facets, followed by diffusion to {100} facets (Figure 2a-I).^32,33^ Further, the ultimate configuration of C-Au/4MBA@Ag NCs at varying pH values was investigated (Figure 2a-II). In acidic or weak alkaline conditions, the amount of NaOH is relatively minor, resulting in the rate of CRR being much slower than that of GRR. The Au/4MBA@Ag NCs exhibit cavity NCs due to one Au^3+^ replacing three Ag+ in the weak CRR, similar to those reported in the literature.^34^Figure 2b displays the various configurations of C-Au/4MBA@Ag NCs. The concave configuration is observed when the pH reaches 9. However, there still exists some NCs with holes in the {100} facets (Figure S7). The NCs with holes nearly disappear when the pH rises to 10, forming large-curvature Au/4MBA@Ag@Au–Ag NCs (LC-Au/4MBA@Ag NCs). When the pH rises to 11, the curvature gradually decreases as the rate of CRR increases, resulting in a slight curvature of Au/4MBA@Ag@Au–Ag NCs (SC-Au/4MBA@Ag NCs). As the pH increases to 12, the concave configuration gradually fades, eventually forming Au/4MBA@Ag@Au–Ag NCs with a cubic Au–Ag alloy frame (CF-Au/4MBA@Ag NCs). To compare the changes more accurately in the curvature, we took the vertical distance from the lowest point of the curved surface to the plane as a quantitative standard. The distance of LC-Au/4MBA@Ag NCs is nearly 4 nm, and the distance of SC-Au/4MBA@Ag NCs is 2 nm. Besides, the distribution map of Ag elements also clearly reveals the concave structure, and the Au element is found at the edge of the NCs, as displayed in the EDS result of typical concave NCs (Figure 2c). Figure S8 depicts the X-ray diffraction (XRD) pattern of C-Au/4MBA@Ag NCs similar to Au and Ag standard XRD patterns. The strong peaks of (111), (200), and (220) indicated that the face-centered cubic (fcc) C-Au/4MBA@Ag NCs was formed. As we mentioned earlier, the GRR causes holes on the {100} facets of the cubic shell, and the size of these holes affects the position of the UV absorption peak. Figure 2d,e compares the peak position of the main UV absorption peak of C-Au/4MBA@Ag NCs formed under various pH conditions, showing that it blue-shifts as the pH increases. This phenomenon can be attributed to the fact that the holes on the Au/4MBA@Ag NCs become fewer and smaller as the co-reduction reaction accelerates, which is consistent with the previous literature report.^33^ Additionally, the width of the UV peak gradually decreases as the pH increases, and the absorption peak at 340 nm reflecting the high-order plasma mode of the NCs becomes more prominent. These findings demonstrate the successful and controllable synthesis of the proposed C-Au/4MBA@Ag NCs.

### SERS Performance of C-Au/4MBA@Ag NCs

#### SERS Sensitivity of C-Au/4MBA@Ag NCs

**Figure 3:**
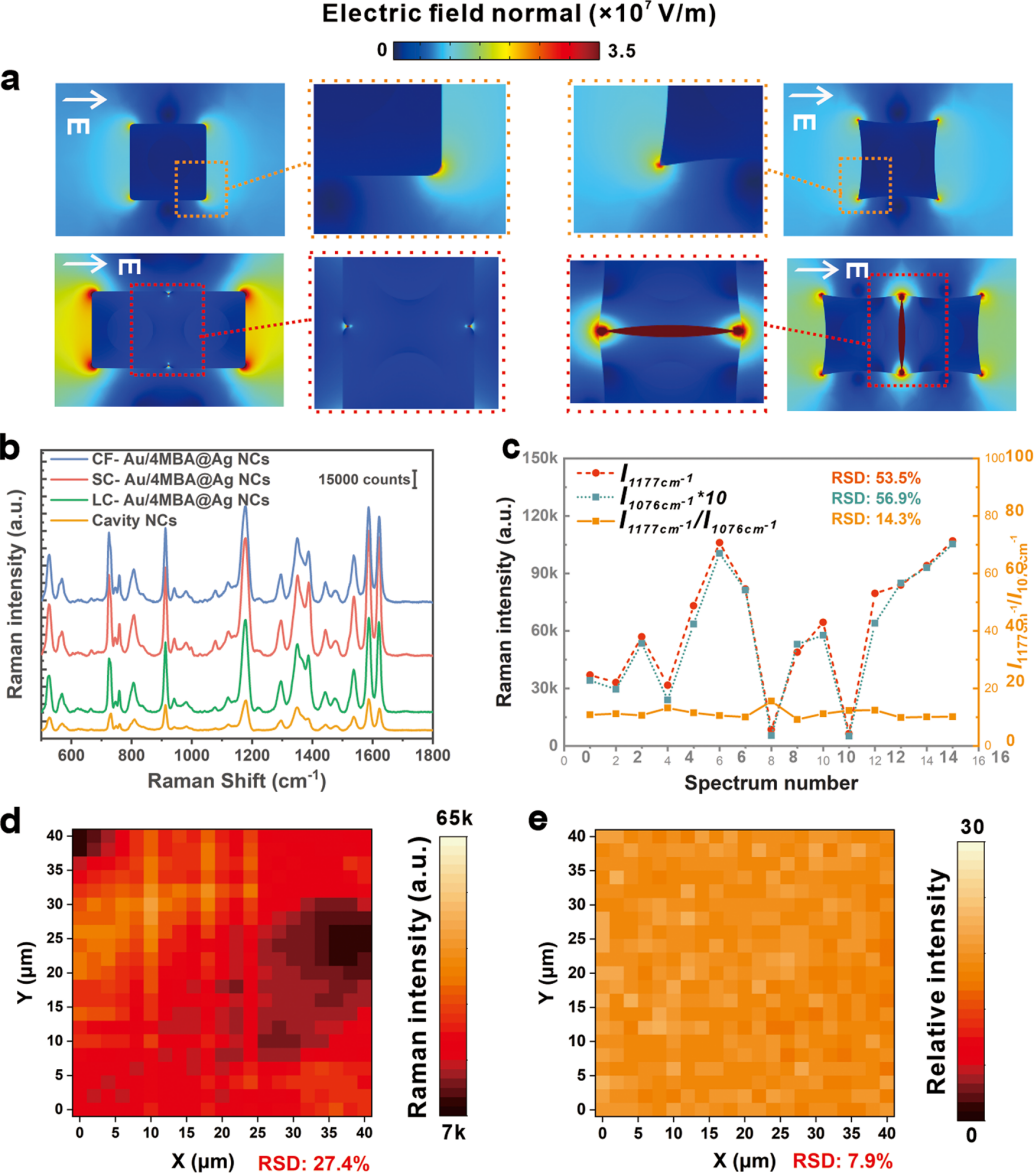
(a) Simulation of localized electric field distribution of Au@Ag NCs, concave Au@Ag NCs, and corresponding enlarged view. (b) Typical SERS spectra of CV with cavity NCs, LC-Au/4MBA@Ag NCs, SC-Au/4MBA@Ag NCs, and CF-Au/4MBA@Ag NCs as SERS substrates. (c) SERS intensities of the CV characteristic peak (1177 cm^–1^) and IS peak (1076 cm^–1^) as well as their intensity ratios across the randomly selected 15 spots when evaporating SC-Au/4MBA@Ag NCs/CV on a silicon wafer. (d, e) The mapping results of SERS signals of SC-Au@Ag NCs/CV (d) and the calibrated SERS signal of SC-Au/4MBA@Ag NCs/CV (e) evaporated on hydrophobic paper.

The concave configuration of the NCs results in sharper eight corners and a stronger EMF than traditional NCs, as shown in Figure 3a, ultimately improving the sensitivity. Additionally, the nanogap between concave facets creates more “hotspots,” leading to further sensitivity improvement. All simulation models were established using the finite element method in COMSOL Multiphysics software. To identify the C-Au/4MBA@Ag NCs with the most effective SERS performance, we conducted a comparison of the IS signal and enhancement effect on CV molecules (1 × 10^–7^ M) for C-Au/4MBA@Ag NCs with varying curvatures (Figure 3b). The full spectra are shown in Figures S9 and S10. The concave configuration has a significantly improved SERS intensity, especially in SC-Au/4MBA@Ag NCs. However, it is worth noting that the absolute intensity of the LC-Au/4MBA@Ag NCs is slightly decreased. This can be attributed to the C-Au/4MBA@Ag NCs being core–shell NPs, and the thickness of the Ag shell directly influences the absolute intensity. During the synthesis process of LC-Au/4MBA@Ag NCs, the GRR rate is still dominant to etch the Ag shell thickness, finally further leading to a slight weakening of the IS and characteristic signals. The concave configuration is essentially absent at a pH greater than 12, resulting in the sensitivity of these structures not being comparable to that of SC-Au/4MBA@Ag NCs. SC-Au/4MBA@Ag NCs possess both a powerful enhancement effect and stable IS peaks, enabling the dual functions of signal self-calibration and enhancement to be achieved. As a result, SC-Au/4MBA@Ag NCs were selected for subsequent studies.

#### SERS Reliability of C-Au/4MBA@Ag NCs

To ensure the reliability of the SC-Au/4MBA@Ag NCs in the SERS detection process, we conducted a systematic investigation by randomly selecting 15 points and examined the SERS spectra. As shown in Figure 3c, we summarized the absolute intensity of the CV characteristic peak and IS peaks to facilitate the analysis. The peaks at 1177 and 1076 cm^–1^ were used as a quantitative peak and an IS peak, respectively, because of their high intensity and noninterference with other peaks. Figure 3c reveals that the quantitative peak intensity varies greatly over 15 points, with a relative standard deviation (RSD) value of up to 53.5%. However, the IS peak displays a floating trend similar to that of the quantitative peak (RSD 56.9%). Therefore, the RSD of the relative intensity of the quantitative peak to IS peaks decreased significantly to 14.3%. These findings indicate that the incorporation of the IS peak can effectively calibrate the flotation of SERS signals and improve the reliability of SERS detection. However, CTAC hinders the self-assembly of SC-Au/4MBA@Ag NCs to fabricate the nanogap and the probe molecules from approaching them (Figure S11). Although the long chain molecules only remain few after washing, it still has some effect on the distribution of the probe molecules (Figure S12).^35^ Our prediction suggests that by concentrating SC-Au/4MBA@Ag NCs and CV molecules, the RSD can be further reduced, and sensitivity can be improved. To validate this prediction, we used hydrophobic paper instead of silicon wafers for SERS detection. Consequently, the SERS signals in a mapping region of 40 μm × 40 μm with a scanning step of 2 μm were recorded (Figure S13). The absolute intensity of the quantitative peak and corresponding relative intensity based on SC-Au/4MBA@Ag NCs, the RSD value of the mapping was calculated, which dramatically dropped from 27.4 to 7.9%, as shown in Figure 3d,e. These findings clearly demonstrate that the signal self-calibration capability of the proposed SC-Au/4MBA@Ag NCs can significantly enhance the reliability of SERS detection. All following experiments use hydrophobic paper as support.

#### Accurate Quantitative Detection Based on SC-Au/4MBA@Ag NCs

**Figure 4:**
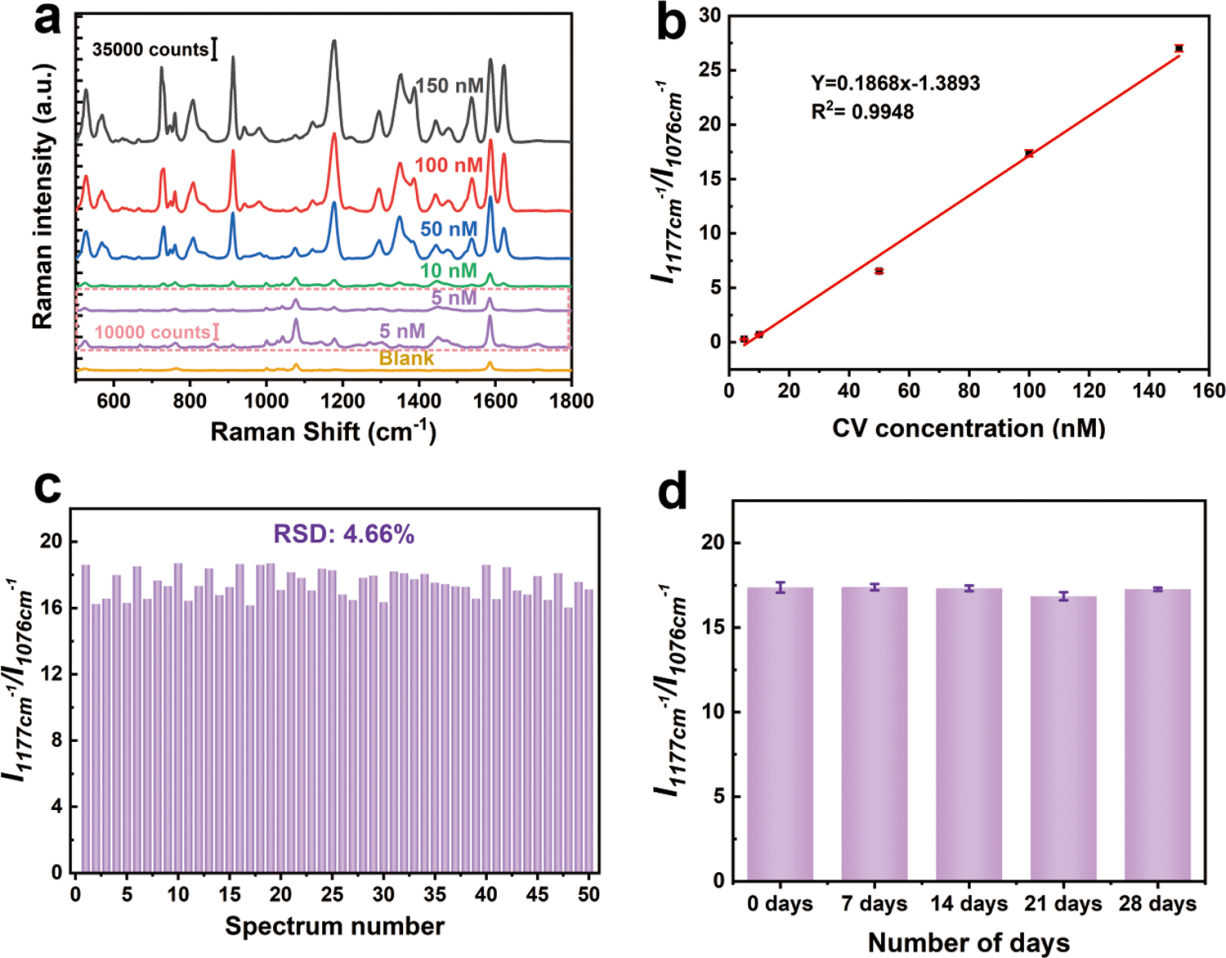
(a) Typical SERS spectra of SC-Au/4MBA@Ag NCs/CV with different CV concentrations. (b) Relationships between the relative SERS intensity of the CV characteristic peak and the logarithm of CV concentration. (c) Relative SERS intensities of the CV characteristic peak across 10 batches based on the SC-Au/4MBA@Ag NCs. (d) Relative SERS intensities of the CV characteristic peak in long-term stability examinations.

To substantiate the sensitivity of our method, distinct SERS spectra of CV molecules at different concentrations were scrutinized. Figure 4a displays typical SERS spectra of CV molecules at diverse concentrations with SC-Au/4MBA@Ag NCs. The peak intensity is observed to decrease with decreasing concentration, while the absolute intensity of the IS peak remains relatively unaffected. The correlation between the CV concentration and the relative intensity of the characteristic peak was examined to evaluate the feasibility of quantitative analysis. Figure 4b shows that the relative intensity (*I*_1177cm_^–1^/*I*_1076cm_^–1^) increases linearly with CV concentration, and this relationship exhibits an excellent linearity of over 0.99. Furthermore, with the concentration of CV as low as 5 × 10^–9^ M, the quantitative peak can still be clearly distinguished. At the same time, the EF values of SC-Au/4MBA@Ag NCs and Au/4MBA@Ag NCs on different supports are calculated in Figure S14. The SC-Au/4MBA@Ag NCs/silicon wafer has an EF of 1.3 × 10^10^, and it is 3.6-fold lower than that on the hydrophobic paper. To confirm that such high EF can be attributed to the “crescent arc” configuration of SC-Au/4MBA@Ag NCs, the control experiment was designed. In the experiment, the unlabeled crescent arc facets Au@Ag NCs (C-Au@Ag NCs) and unlabeled Au@Ag NCs (Au@Ag NCs) were synthesized. 1 ×10^–7^ M 4MBA was first mixed with the C-Au@Ag NCs. After 30 min, the mixture was washed with water and then added with 5 × 10^−7^ M CV solution. After repeating the “incubate–wash” step, 20 μL of the solution was taken and dried on a silicon wafer. Three samples were randomly selected, a total of 100 points, to collect the SERS spectrum, and then the ratio of *I*_4MBA_ to *I*_CV_ was calculated. It is important that 4MBA is not the internal standard and is absorbed on the surface of the nanostructure. As the control experiment, Au@Ag NCs also underwent the same operation. 4MBA has a thiol group that can be tightly absorbed on the surface of the nanostructure. Therefore, we assume that the amount of 4MBA on the surface of the two nanostructures is the same. In theory, a large amount of 4MBA molecules occupied the “hotspot” region and the CV molecules hardly entered it. The SERS signals of CV were used to indirectly estimate the amount of CV on the surface or within the nanoscale gaps (“hotspot” region). Given the differences in enhancement between the two nanostructures, comparing the absolute intensity of characteristic peaks alone is insufficient to reflect the number of molecules entering the nanogaps. Thus, the ratio was used to quantify the amount of CV. We assume that the “crescent arc” facets have a higher ratio value than traditional Au@Ag NCs, which represents that much more CV molecules enter the nanogaps. Figure S15 clearly depicts that the ratio of concave NCs is much higher than that of traditional NCs. These observations suggest that the enhancement effect of the SC-Au/4MBA@Ag NCs/CV system is primarily due to the SC-Au/4MBA@Ag NCs, which generates controllable interparticle nanogaps to offer large depositing spaces for analytes and improve authoritative SERS signals’ enhancement. Moreover, the concentration effect of the hydrophobic paper is also a key factor, as it further produces many more and narrower nanogaps, as shown in Figure S16. Thus, the SC-Au/4MBA@Ag NCs can satisfy the demanding requirements of high sensitivity.

Consistency in SERS signals across batches and the stability of the SERS substrate over the long term are critical aspects for accurate quantification. To investigate this performance, the SERS spectra of 1 × 10^–7^ M CV from 10 batches were analyzed. The relative intensity of various batches is combined to form Figure 4c. The results demonstrate that the RSD between batches is as low as 4.66%, indicating excellent reproducibility and thereby confirming the high reliability of SC-Au/4MBA@Ag NCs. In Figure 4d, changes in the SC-Au/4MBA@Ag NC SERS system were monitored during long-term storage (28 days). Notably, even after 28 days of storage, the SC-Au/4MBA@Ag NC SERS system showed consistent results, demonstrating its long-term stability. These above findings serve to fully demonstrate the excellent reliability, sensitivity, reproducibility, and long-term stability of the SC-Au/4MBA@Ag NCs system, which is expected to be applied to practical SERS quantitative detection.

#### Proposed Method for Pesticide Residue Detection

**Figure 5:**
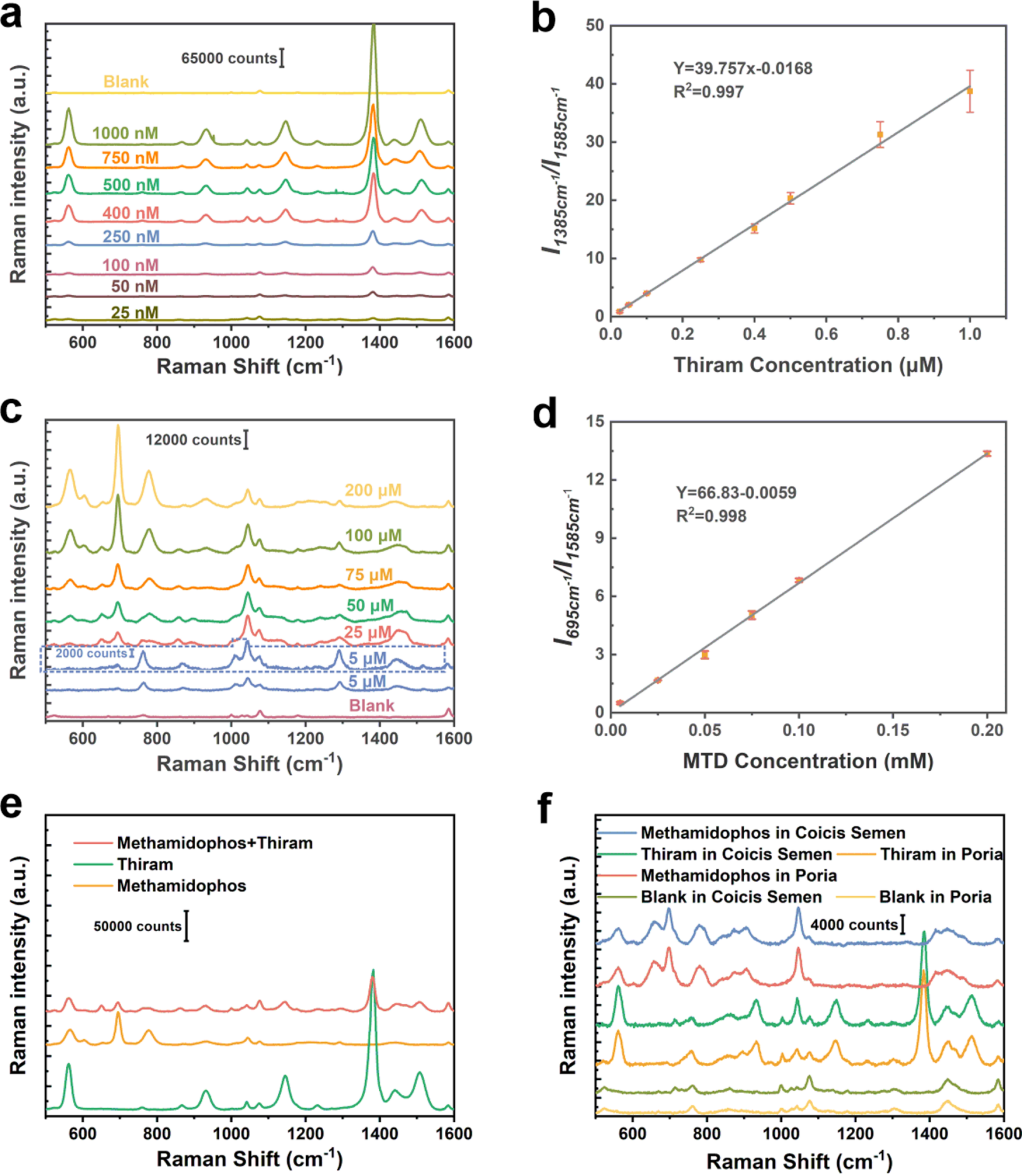
(a, c) Typical SERS spectra of thiram (a) and methamidophos (c) with different concentrations. (b, d) Corresponding relationship between the relative SERS intensity of thiram (b) and methamidophos (d) characteristic peak and concentration. (e) The typical SERS spectra of the thiram and methamidophos mixture solution. (f) The typical SERS spectra of thiram and methamidophos spiked in two herbal plants.

An investigation was undertaken by using thiram as a representative pesticide to assess the practical potential of the proposed SC-Au/4MBA@Ag NCs system. The SC-Au/4MBA@Ag NCs were incubated at different concentrations of thiram. Typical spectra for the SC-Au/4MBA@Ag NCs and thiram are illustrated in Figure 5a, with concentrations ranging from 1 × 10^–6^ M to 2.5 × 10^–8^ M. While the characteristic peaks of 4MBA (1076 and 1585 cm^–1^) fall in the Raman active region, they still remain distinguishable from the characteristic peaks of thiram. The peaks located at 1385 and 1585 cm^–1^ were respectively designated as the quantitative peak and the IS peak. The results indicate a positive linear relationship between the relative intensity of the quantitative peak observed at 1385 cm^–1^ and the concentration of thiram, and the correlation coefficient was 0.997 (Figure 5b). Furthermore, the limit of detection (LOD) can be determined by the equation LOD = 3*S*_b_/*m*, where *S*_b_ represents the standard deviation of the blank sample and *m* denotes the slope of the corresponding calibration curve. The LOD of thiram was calculated to be 2.9 × 10^–9^ M, and such a low LOD is attributed to the well-designed concave configuration of SC-Au/4MBA@Ag NCs. The high correlation coefficient and remarkably low LOD demonstrate the properties of our SC-Au/4MBA@Ag NCs in detecting pesticide residues. To prove that such high sensitivity is not limited to thiram, a highly toxic organophosphorus pesticide, methamidophos, was selected as a probe molecule, and characteristic peaks are observed at 562, 652, 695, and 774 cm^–1^, as shown in Figure 5c. The peaks at 695 and 1585 cm^–1^ were selected as the quantitative peak and IS peak, respectively, and the LOD was calculated as 1.5 × 10^–7^ M. The correlation coefficient is 0.998, indicating that our proposed system has high sensitivity and reliability (Figure 5d). The SC-Au/4MBA@Ag NCs have significantly better LODs compared to traditional core–shell-based SERS substrates due to their concave configuration and internal nanogaps. These results demonstrate the potential of the proposed SC-Au/4MBA@Ag NC SERS substrate for practical applications. Table S1 illustrates that the SC-Au/4MBA@Ag NC system is more reliable and sensitive than similar methods for detecting thiram and other contaminant molecules.

#### Real Sample Analysis

Multiple pesticides are usually sprayed on grains, vegetables, and special herbal plants to prevent infestations and bolster crop yield. Thus, SERS substrates with high sensitivity and reliability are crucial for accurately discerning each pesticide’s SERS signal from the signals of other interfering components. We first verified the detection capability of our proposed SERS system for the detection of mixed pesticides. Figure 5e depicts that the characteristic peaks located at 695 and 1385 cm^–1^ belonging to methamidophos and thiram, respectively, can be clearly distinguished. To further evaluate our proposed SERS system, different concentrations of thiram and methamidophos were mixed with Coicis Semen and Poria. The SERS spectra of thiram and methamidophos in Coicis Semen and Poria are displayed in Figure 5f. It is found that the absolute intensity of the characteristic peaks of pesticides extracted from herbs is significantly reduced due to the matrix effect. However, the inherent IS characteristic peak is also correspondingly reduced, which leads to the relative intensity being similar to that of the standard solution. To evaluate the detection accuracy further, we calculated the relative intensity from spiked herbal plants and compared it with a standard solution. The concentrations of methamidophos and thiram were calculated and are presented in Table S2. The standard concentrations are consistent with the spiked concentrations, and the average detection accuracy is up to 96%. The result provides compelling evidence that this system demonstrates remarkable reliability and sensitivity. The LODs for herbal plants are as low as 0.002 and 0.05 mg/kg, which aligns with the regulations prescribed by the Chinese Pharmacopoeia.

## Conclusions

In conclusion, we demonstrated a controlled substrate synthesis strategy to enable reliable and sensitive SERS detection by sculpturing the curvature of C-Au/4MBA@Ag NCs. The bifunctional IS in the core–shell NCs could alter the configuration of the shell and calibrate SERS signal flotation to boost reliability. Additionally, the “crescent arc” facets generate controllable interparticle nanogaps to offer “hotspots” and large depositing spaces for analytes, enhancing authoritative SERS signals. The optimized C-Au/4MBA@Ag NCs exhibited an EF as high as 4.8 × 10^10^ and an ultralow RSD of 7.9% using CV as a probe molecule. The pesticide residues in different herbal plants could also be quantitatively detected, with an average detection accuracy of up to 96%. This study not only provides an effective strategy to modulate the surface structure simply by changing the chemical reaction rates but also systematically reveals the synergistic effect of small molecules as IS and surfactants on metal morphology as the underlying mechanism. More generally, this study sheds new light on the relationship between surface topography and SERS performance and paves the way for the widespread application of unique nanocube substrates in surface-enhanced Raman spectroscopy.

## Supporting Information Available

Additional experimental details; materials; methods; figures; and tables, as mentioned in the text (PDF)
