# Supplementary material for: Plasmonic Nanocubes with a Controllable “Crescent Arc” Facet: Tunable Hotspot Engineering for Highly Reliable and Sensitive SERS Detection
Source: Anal Chem. 2024 Oct 17;96(43):17453–62. doi: 10.1021/acs.analchem.4c05334 (PMC11525925; doi:10.1021/acs.analchem.4c05334)
Supplement: Supplementary file 1 — ac4c05334_si_001.pdf [file ac4c05334_si_001.pdf]

SUPPORTING INFORMATION

Plasmonic nanocubes with controllable "crescent arc" facet: a tunable hotspot engineering for highly reliable and sensitive SERS detection

Ting Wang[†], Jinchao Wei[†], Zehua Cheng, Mai Luo, Liang Zou, Lele Zhang, Mei Zhang [*], Peng Li [*]

Ting Wang – State Key Laboratory of Southwestern Chinese Medicine Resources, School of Pharmacy, Chengdu University of Traditional Chinese Medicine, Chengdu 611137, China; State Key Laboratory of Quality Research in Chinese Medicine, Macau Centre for Research and Development in Chinese Medicine, Institute of Chinese Medical Sciences, University of Macau, Macao 999078, China

Jinchao Wei – State Key Laboratory of Quality Research in Chinese Medicine, Macau Centre for Research and Development in Chinese Medicine, Institute of Chinese Medical Sciences, University of Macau, Macao 999078, China

Zehua Cheng – State Key Laboratory of Quality Research in Chinese Medicine, Macau Centre for Research and Development in Chinese Medicine, Institute of Chinese Medical Sciences, University of Macau, Macao 999078, China

Mai Luo – State Key Laboratory of Quality Research in Chinese Medicine, Macau Centre for Research and Development in Chinese Medicine, Institute of Chinese Medical Sciences, University of Macau, Macao 999078, China

Liang Zou – School of Food and Biological Engineering, Chengdu University, Chengdu 610106, China

Lele Zhang – School of Food and Biological Engineering, Chengdu University, Chengdu 610106, China

Mei Zhang – State Key Laboratory of Southwestern Chinese Medicine Resources, School of Pharmacy, Chengdu University of Traditional Chinese Medicine, Chengdu 611137, China

Peng Li – State Key Laboratory of Quality Research in Chinese Medicine, Macau Centre for Research and Development in Chinese Medicine, Institute of Chinese Medical Sciences, University of Macau, Macao 999078, China

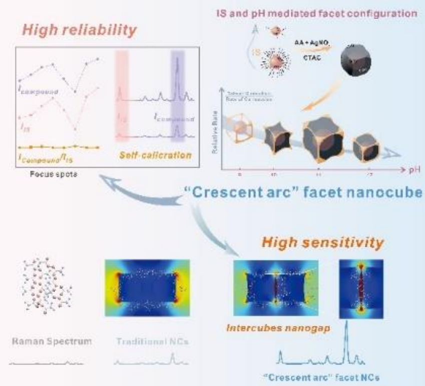

[†] Ting Wang, and Jinchao Wei contributed equally to this work.
* Corresponding authors:
E-mail addresses: pli1978@hotmail.com (P. LI); zhangmei63@cdutcm.edu.cn (M. ZHANG)

EXPERIMENTAL SECTION

Synthesis of C-Au/4MBA@Ag NCs

***Synthesis of Au-4MBA NPs*:** Au NPs were synthesized by sodium borohydride reduction[1]. Briefly, the Au NPs solution was prepared by repeating the seed growth method until 35 nm, followed by mixing it with different concentrations of 4MBA solution (5×10^{-5} M, 5×10^{-6} M, 5×10^{-7} M, 1×10^{-7} M, and 1×10^{-8} M) and incubating it for one hour.

***Synthesis of Au/4MBA@Ag NPs*:** 13 mL Au-4MBA NPs were added to 18 mM CTAC solution, heated at 65°C for 20 min in a water bath, and then 5 mL of 2 mM $AgNO_3$ and 50 mM AA solution were added simultaneously, and stirred for 3h.

***Synthesis of C-Au/4MBA@Ag NCs with controlled curvature*:** 1 mL of 0.1 mM $HAuCl_4$ was added dropwise to a mixed solution consisting of Au/4MBA@Ag NCs solution, 100 mM CTAC, 100 mM AA and NaOH. The pH of the solution was controlled by varying the amount of NaOH. And then, centrifuged and washed 3 times immediately after addition to obtain C-Au/4MBA@Ag NCs solution, which was concentrated 5 times for subsequent SERS detection.

Chemicals and Materials

Chloroauric acid hydrate ($HAuCl_4{\cdot}4H_2O$, 99.99%), Sodium borohydride ($NaBH_4$, 98%), silver nitrate ($AgNO_3$, 99.85%) and sodium hydroxide (NaOH, 97%) were purchased from Sigma-Aldrich (St. Louis, MO, U.S.A.). Ascorbic acid (AA, $C_6H_8O_6$, 99.99%) and thiram ($C_6H_{12}N_2S_4$, 97%) were obtained from Aladdin Chemical Co. Ltd, China. 4-Mercaptobenzoic acid (4MBA, $HSC_6H_4CO_2H$, 99%), Crystal violet (CV, $C_{25}N_3H_{30}Cl$, 98% and Cetyltrimethylammonium chloride (CTAC, $CH_3(CH_2)_{15}N(Cl)(CH_3)_3$, 99%) were acquired by Macklin Biochemical (Shanghai, China). Methamidophos ($C_2H_8NO_2PS$, 98.5%) was obtain from Dr. Ehrenstorfer Gmbh (Augsburg, Germany). Krytox GPL 105 lubricant was collected from DuPont (New York, USA). Polytetrafluoroethylene (PTFE) filter papers with a pore size of 0.1 μm were purchased from Haining Yibo Filter Equipment Co., Ltd. (Zhejiang, China). Ultrapure Milli-Q water (18.2 MΩ· cm) was used to make up all experimental solutions.

Instruments

All Raman spectra were collected by confocal Raman microscope (InVia, Renishaw, UK). High-resolution transmission electron microscopy (HRTEM) and scanning transmission electron microscopy-energy dispersive X-ray spectroscopy (STEM-EDS) elemental mapping images were obtained with transmission electron microscopy (Talos F200X, FEI, Massachusetts, USA) at an accelerating voltage of 200 kV. Scanning electron microscopy (SEM) images were captured by field emission scanning electron microscopy (FESEM, Zeiss Sigma, Germany). UV-Vis absorption spectra were recorded on a DR 6000 UV-Vis spectrophotometer (HACH, Colorado, USA). The syringe pump was KDS Legato 270 (KD Scientific, Inc., USA).

Sample preparation and SERS examination

The CV, methamidophos, and thiram were first dissolved with certain concentrations as pre-solutions. Then 5 μL pre-solution was mixed with 45 μL C-Au/4MBA@Ag NCs solution, followed by the incubation process for 30 min. The concentration of relevant analytes in the mixed solution was recalculated. Finally, 20 μL of the mixed solution was dropped and naturally evaporated on the silicon wafer or the hydrophobic paper for corresponding SERS examinations. For the mixture of pesticides and herbal plant samples, 5 mL pre-solution of the paraquat and thiram were directly mixed with 2 g Coicis Semen and Poria, respectively. After shaking for 1 min, the mixture was moved onto a poly (ether sulfone) ultrafiltration membrane (with a mesh dimension of 0.22 μm) to obtain the supernatant. Such supernatants served as the pre-solutions of the pesticide/herbal plant samples for the SERS examination with other parameters unchanged as mentioned above. All the SERS spectra were obtained with the excitation wavelength of 785 nm, power density of 0.05% (1.5 mW), objective lens of 50× and exposure time of 10 s.

COMSOL model establishment for the simulation of localized electric filed distributions

The models were established in a three-dimensional structure using COMSOL Multiphysics software. The sphere with diameter of 30 nm was employed as the Au core. The 20 nm rounded cubic shell and concave cubic shell were utilized to form the Au@Ag NCs and C-Au@Ag NCs, respectively. The Au@Ag NCs and C-Au@Ag NCs structure were obtained by the Boolean operation. The detailed geometric configuration of the C-Au@Ag NCs and Au@Ag NCs can be referred to Figure 3a. All the geometric structures were enclosed in an air domain. The typical excitation power of 1 mW and electric mode field of 1V/m were employed for convenient simulation. For Au, Ag and air domain, the refractive index and extinction coefficient were all chosed from material liabrary. The largest mesh size was 2 and smallest was 0.01 for Au@Ag NCs and C-Au@Ag NCs structure. The other parameters such as electrical conductivity and relative permeability were set as default. The excitation wavelength was set as 785 nm in accordance with the experiments.

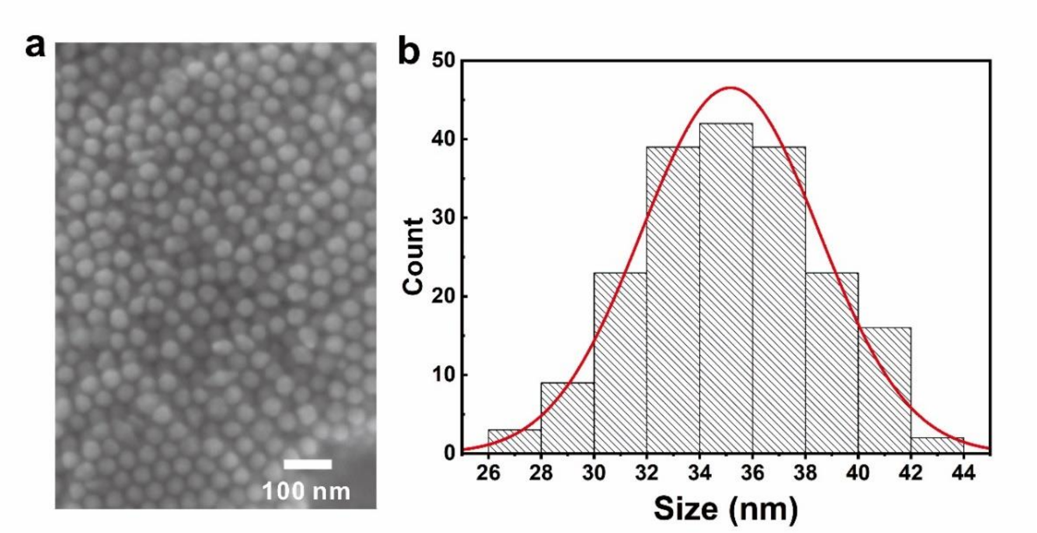

Figure S1. a) Typical SEM images of the pristine Au NPs. b) Size statistics of the Au NPs.

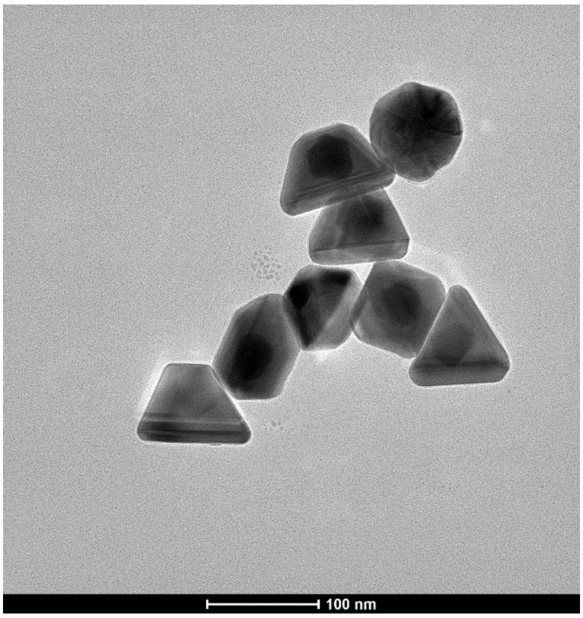

Figure S2. The typical TEM image of Au/4MBA@Ag NPs with 5×10^{-6} M 4MBA.

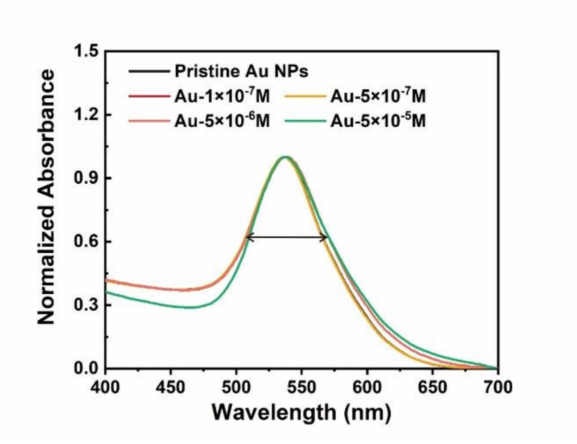

Figure S3. The UV-Vis spectra of Au NPs with different concentration 4MBA.

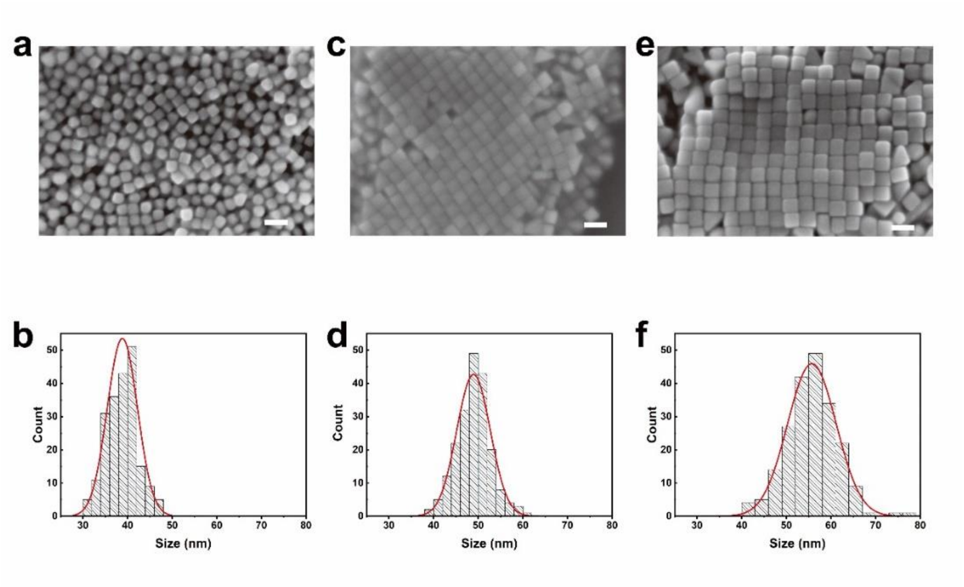

Figure S4. The typical SEM images and particle size statistics of different amount of $AgNO_3$. a-b) 0.167 mM $AgNO_3$. c-d) 0.333 mM AgO_3. e-f) 0.375mM $AgNO_3$. The scale bar is 100nm.

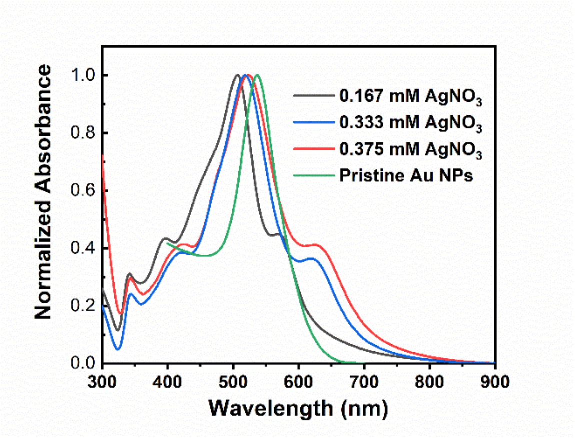

Figure S5. UV/Vis absorption spectra of Au/4MBA@Ag NCs with different amount of $AgNO_3$.

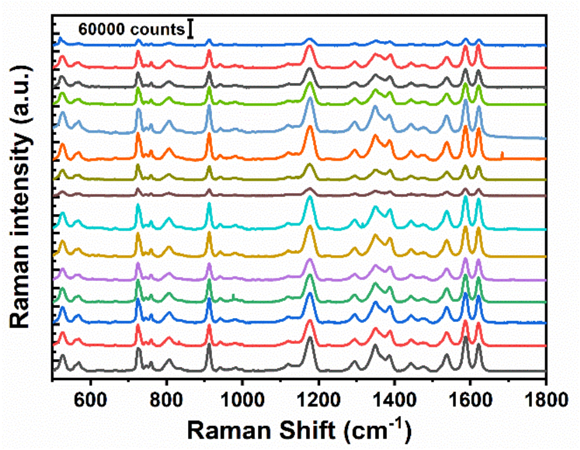

FigureS6. SERS spectra of Au/4MBA@Ag NCs (1×10^{-8} M 4MBA)/CV.

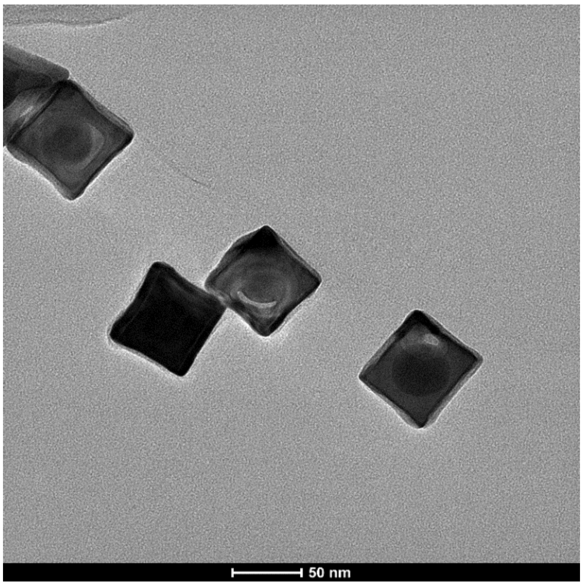

Figure S7. The typical TEM image of C-Au/4MBA@Ag NCs in pH 9.

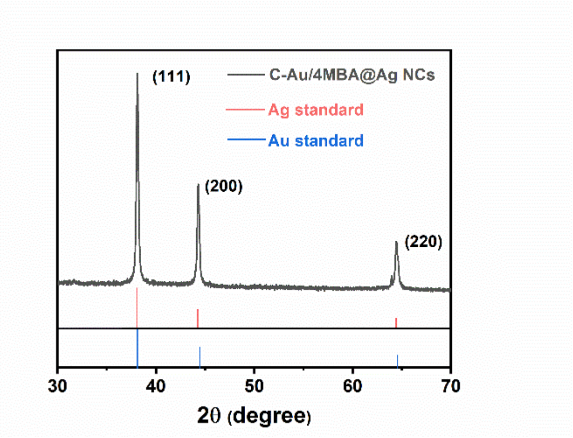

Figure S8. XRD pattern of C-Au/4MBA@Ag NCs.

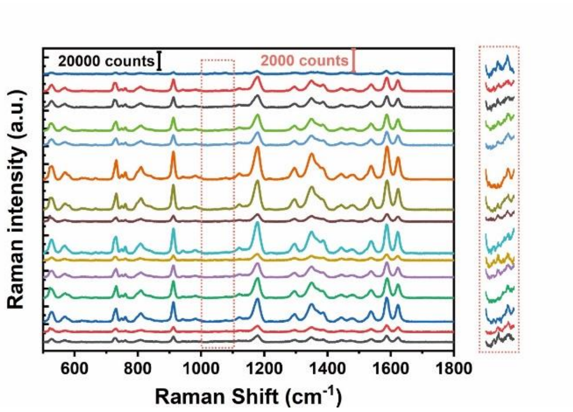

Figure S9. SERS spectra of the cavity NCs/CV across the randomly selected 15 spots on the silicon wafer.

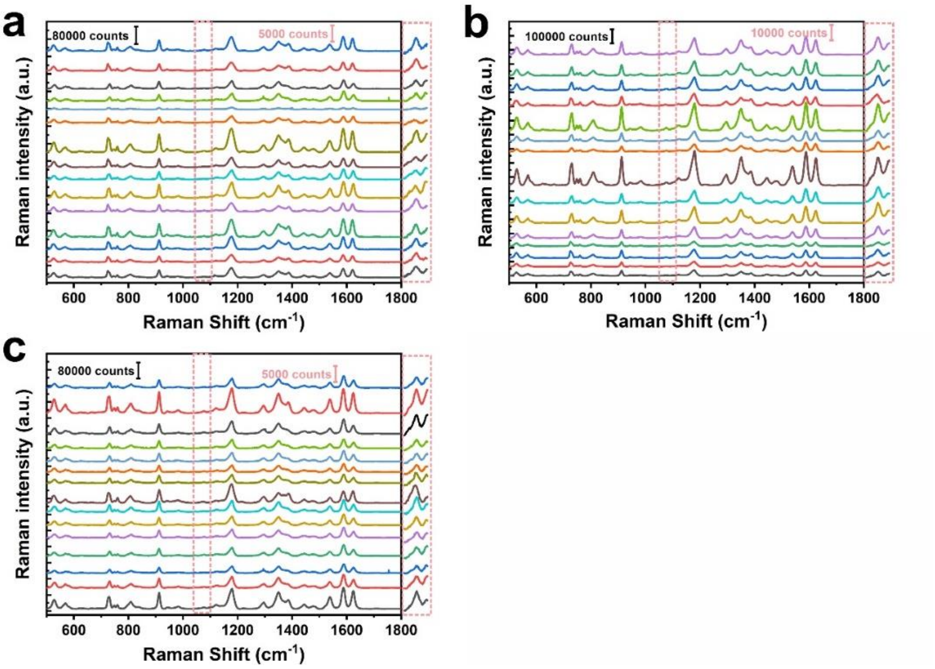

Figure S10. SERS spectra of the LC-Au/4MBA@Ag NCs/CV (a), SC-Au/4MBA@Ag NCs/CV(b) and CF-Au/4MBA@Ag NCs/CV (c) across the randomly selected 15 spots on the silicon wafer.

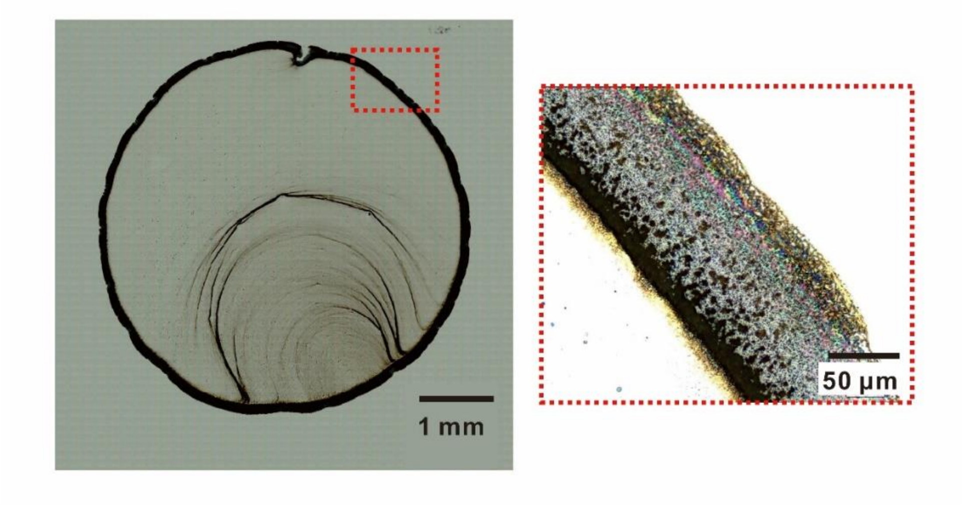

Figure S11. The optical images of the SC-Au/4MBA@Ag NCs with CTAC distributed on the silicon wafer.

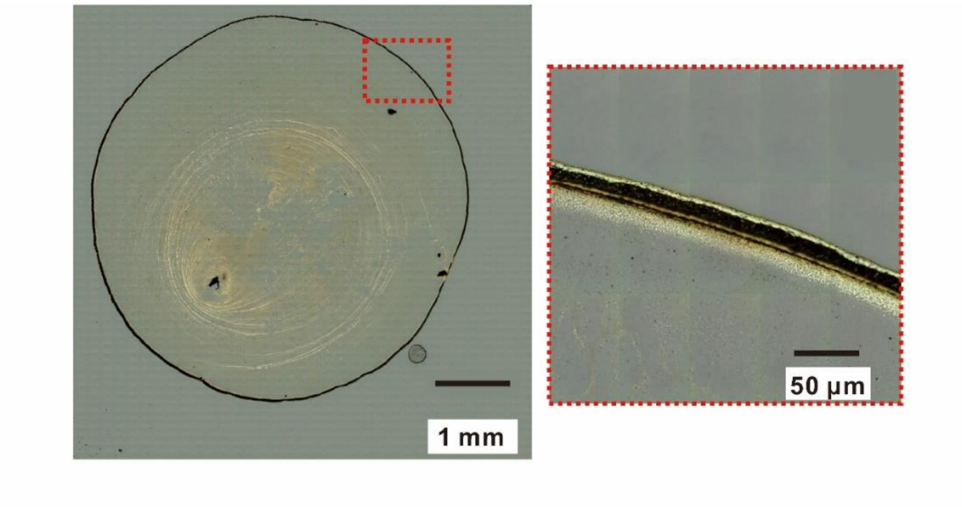

Figure S12. The optical images of the cleaned SC-Au/4MBA@Ag NCs distributed on the silicon wafer.

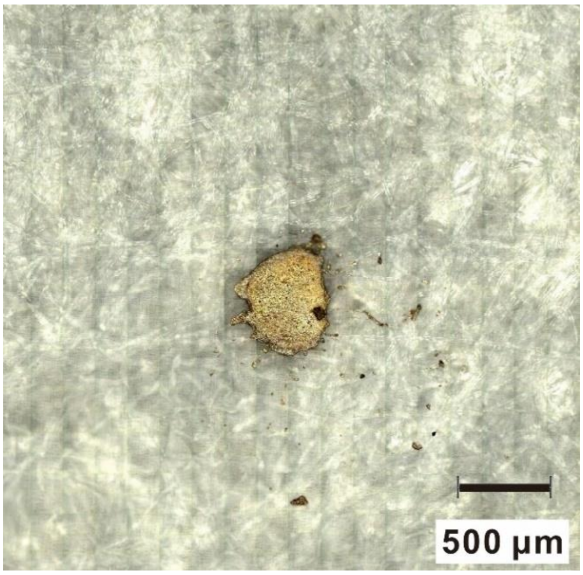

Figure S13. The optical images of the cleaned SC-Au/4MBA@Ag NCs distributed on the hydrophobic paper.

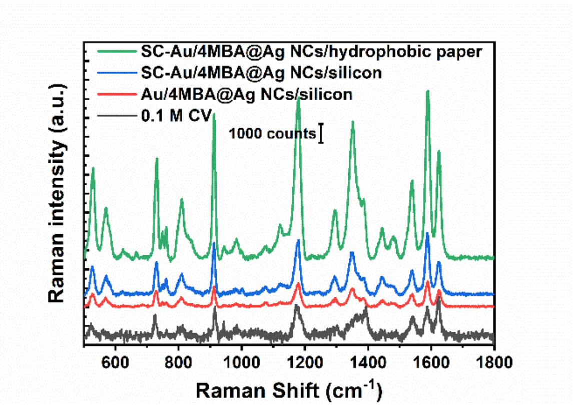

Figure S14. The SERS spectra of Au/4MBA@Ag NCs/CV (red line) and SC-Au/4MBA@Ag NCs/CV (bule line) on the silicon wafer as well as the Raman spectrum of 0.1 mol/L CV solution (black line). The green line was SC-Au/4MBA@Ag NCs on the hydrophobic paper. The concentration of CV in Au/4MBA@Ag NCs and SC-Au/4MBA@Ag NCs systems was 10^{-7} mol/L.

The EF was calculated by the widely used method [2-4]. $EF=(\boldsymbol{I}_{SERS}/\boldsymbol{C}_{SERS})/(\boldsymbol{I}_{Raman}/\boldsymbol{C}_{Raman})$, where $\boldsymbol{I}_{SERS}$ and $\boldsymbol{I}_{Raman}$ were the SERS intensity of Au/4MBA@Ag NCs and SC-Au/4MBA@Ag NCs of 10^{-7} mol/L CV and Raman intensity of 0.1 mol/L CV at 1076 cm^{-1}. $\boldsymbol{C}_{SERS}$ *and* $\boldsymbol{C}_{Raman}$ were the concentration of 10^{-7}mol/L CV in Au/4MBA@Ag NCs and SC-Au/4MBA@Ag NCs systems and 0.1 mol/L CV, respectively. The laser density in Au/4MBA@Ag NCs and SC-Au/4MBA@Ag NCs systems was 0.0001% and in 0.1M CV system was 1%.

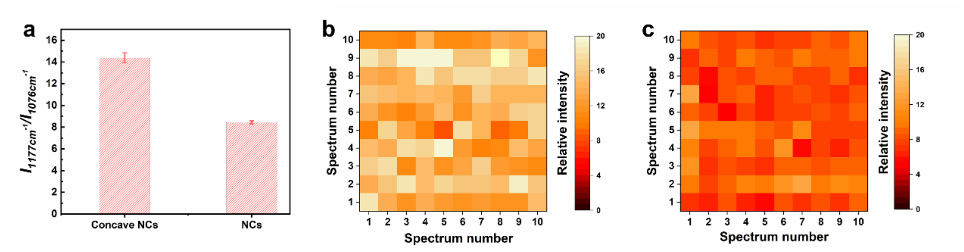

Figure S15. a) The comparison of SERS relative intensity of C-Au@Ag NCs and Au@Ag NCs. b) and c) The mapping results of full data (100 points) of C-Au@Ag NCs and Au@Ag NCs.

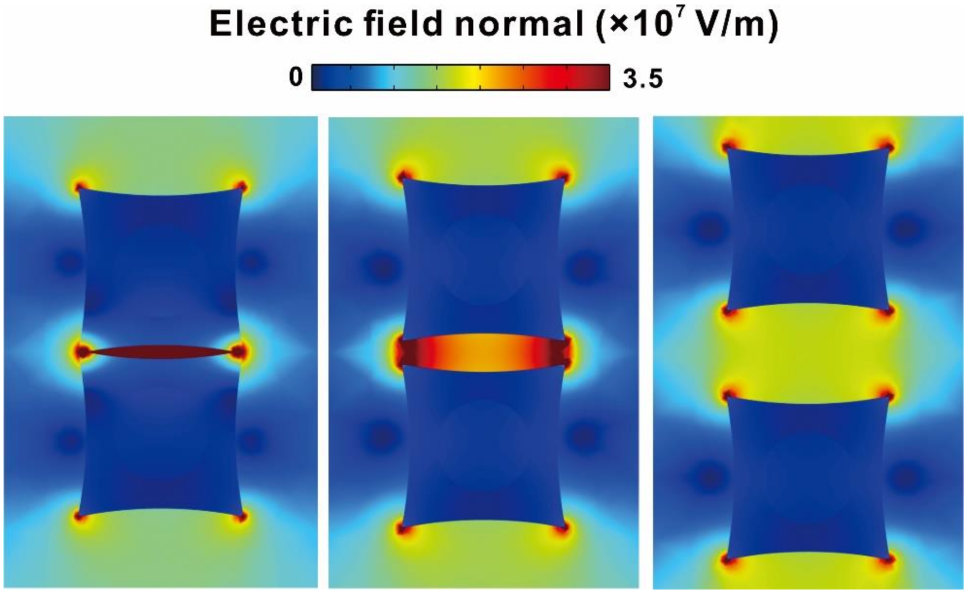

Figure S16. Simulation of localized electric field distribution of two SC-Au@Ag NCs with different distance nanogaps.

Table S1. Comparison of the Concave Au/4-MBA@Ag NCs/hydrophobic paper substrate with other substrates.

No.	Substrate	Internal standard	Analyte	LOD	RSD of SERS mapping (area)	Reference
1	Au@IS@Ag NPs	4-mercaptobezoic acid	Thiram	5×10^{-7} M	9.29%<br>(20 μm×20 μm)	[5]
2	AuNR-bridged Au@Ag	(E)-2-((4-(phenylethynyl)benzylidene)amino)ethanethiol	Thiram	1×10^{-7} M	14.9%<br>(Randomly 20 points)	[6]
3	AuNPs with Semi-Wrapped Prussian Blue	Prussian Blue	Thiram	2.8×10^{-8} M	8.55%<br>(40 μm×40 μm)	[7]
4	SiO_2@Ag-PDMS	PDMS	methylene blue	1×10^{-7} M	17.18%<br>(40 μm×40 μm)	[8]
5	Au@AgNPs decorated 2D Ni-MOF nanosheets	_	Thiram	87.1 μg/L	19.6%<br>(Randomly 25 points)	[9]
6	Au NPs with microplate	-	Thiram	10 ppb	-	[10]
7	Au-Ni nanowires	-	Thiram	29.7 ppb	-	[11]
8	Concave Au/4-MBA@Ag NCs	4-mercaptobezoic acid	Thiram<br>methamidophos	2.9×10^{-9} M<br>1.5×10^{-7} M	7.9%<br>(40 μm×40 μm)	This work

Table S2. Detection of thiram and methamidophos in herbal plants

Analyte	Matrices	Spiked (μM)	Detected (μM)	Accuracy (%)	RSD (%, n=3)
Thiram	Coicis Semen	0.1	0.097	97	3.75
		0.25	0.263	95	1.90
		0.5	0.499	99	4.44
	Poria	0.1	0.095	95	13.52
		0.25	0.242	97	3.84
		0.5	0.501	100	1.52
Methamidophos	Coicis Semen	50	43	87	5.96
		75	78	96	8.28
		100	99	99	5.75
	Poria	50	47	94	5.56
		75	79	94	5.42
		100	102	98	3.43

REFERENCES

(1) Ma, Y.; Li, W.; Cho, E. C.; Li, Z.; Yu, T.; Zeng, J.; Xie, Z.; Xia, Y. Au@Ag Core−Shell Nanocubes with Finely Tuned and Well-Controlled Sizes, Shell Thicknesses, and Optical Properties, *ACS Nano* **2010**, 4 (11), 6725-6734.

(2) Bai, S.; Serien, D.; Hu, A.; Sugioka, K. 3D Microfluidic Surface-Enhanced Raman Spectroscopy (SERS) Chips Fabricated by All-Femtosecond-Laser-Processing for Real-Time Sensing of Toxic Substances. *Adv. Funct. Mater.* **2018**, *28*, 1706262.

(3) Jia, K.; Xie, J.; He, X.; Zhang, D.; Hou, B.; Li, X.; Zhou, X.; Hong, Y.; Liu, X. Polymeric micro-reactors mediated synthesis and assembly of Ag nanoparticles into cube-like superparticles for SERS application. *Chem. Eng. J.* **2020**, *395*, 125123.

(4) Li, M.; Wang, J.-Y.; Chen, Q.-Q.; Lin, L.-H.; Radjenovic, P.; Zhang, H.; Luo, S.-Y.; Tian, Z.Q.; Li, J. F. Background-Free Quantitative Surface Enhanced Raman Spectroscopy Analysis Using Core–Shell Nanoparticles with an Inherent Internal Standard. *Anal. Chem.* **2019**, *91*, 15025-15031.

(5) Lin, S.; Lin, X.; Han, S.; Liu, Y.; Hasi, W.; Wang, L. Flexible fabrication of a paper-fluidic SERS sensor coated with a monolayer of core–shell nanospheres for reliable quantitative SERS measurements. *Anal. Chim. Acta* **2020**, *1108*, 167-176.

(6) Mei, R., Wang, Y., Yu, Q., Yin, Y., Zhao, R., Chen, L. Gold nanorod array-bridged internal-standard SERS tags: from ultra sensitivity to multifunctionality, *ACS Appl. Mater. Interfaces* **2019**, 12(2), 2059-2066

(7) Wang, T.; Ji, B.; Cheng, Z.; Chen, L.; Luo, M.; Wei, J.; Wang, Y.; Zou, L.; Liang, Y.; Zhou, B.; et al. Semi-wrapped gold nanoparticles for surface-enhanced Raman scattering detection. *Biosens. Bioelectron.* **2023**, *228*, 115191.

(8) Ruan, S.; Li, X.; Jiang, T. Hydrophilic-hydrophobic poly (dimethyl siloxane)-based SERS substrate with internal Raman signaling. *Mater. Chem. Phys.* **2020**, *255*, 123582.

(9) Lai, H.; Dai, H.; Li, G.; Zhang, Z. Rapid determination of pesticide residues in fruit and vegetable using Au@ AgNPs decorated 2D Ni-MOF nanosheets as efficient surface-enhanced Raman scattering substrate. *Sens. Actuators B Chem.* **2022**, *369*, 132360.

(10) Zhou, B.; Qu, C.; Du, S.; Gao, W.; Zhang, Y.; Ding, Y.; Wang, H.; Hou, R.; Su, M.; Liu, H. Multi-analyte High-Throughput Microplate-SERS Reader with Controllable Liquid Interfacial Arrays. *Anal. Chem.* **2022**, *94*, 7528-7535.

(11) Karn-orachai, K.; Sanguansap, Y.; Pankleaub, K.; Noppha, O.; Wiriyakun, N.; Kanatharana, P.; laocharoensuk, R. Internal magnetic driven self-assembly of gold-nickel nanowires as SERS substrate for thiram fungicide detection using handheld Raman spectrometer. *Appl. Surf. Sci.* **2020**, *529*, 147236.
